# Identifying Predictors and Measuring Variation in Anticholinergic Burden and Sedative Load for Older Adult Care Home Residents: A Retrospective Cohort Study Using Routinely Collected Data

**DOI:** 10.2147/POR.S618970

**Published:** 2026-10-09

**Authors:** Carl Marincowitz, Richard Jacques, Katherine Zwerger, Graham Martin, Karen Spilsbury, Jennifer Kirsty Burton, Fawn Harrad-Hyde, David Price, Emily Lam, Hilary M Garrett, Suzanne Mason

**Affiliations:** 1 School of Medicine and Population Health, The University of Sheffield, Sheffield, UK; 2 THIS Institute, University of Cambridge, Cambridge, UK; 3 Faculty of Medicine and Health, University of Leeds, Leeds, UK; 4 School of Cardiovascular & Metabolic Health, University of Glasgow, Glasgow, UK; 5 Health Sciences, University of Leicester, Leicester, UK; 6 Optimum Patient Care UK, Cambridge, UK; 7 Patient Public Contributor, Health Data Research UK, London, UK

**Keywords:** anticholinergic burden, sedative load, care homes

## Abstract

**Purpose:**

There over 250,000 care home residents in England and they account for a disproportionate number of hospital admissions. Since 2020, structured medication reviews for care home residents have been mandated in primary care contracts. We used a nationally representative cohort of care home residents to identify predictors of anticholinergic burden and sedative load and measure regional variation in prescribing.

**Patients and Methods:**

Using primary care data from a third of practices in England we derived four 6-month cohorts of over 40,000 care home residents linked to the national regulator’s register of care homes between 01/01/2023 and 31/12/2024. We estimated three-level logistic regression models including resident, postcode district and commissioner (ICB) level factors which predicted high anticholinergic burden and sedative load. We estimated funnel-plots to measure case-mix risk adjusted variation in prescribing at postcode district and commissioner geographical levels.

**Results:**

Significant predictors of anticholinergic burden and sedative load included gender, age, frailty, and comorbidity. We demonstrated some case-mix adjusted variation at ICB and postcode district level (anticholinergic burden 2.3-fold ICB and 3.5-fold variation at district level; sedative load 1.7-fold ICB and 2.7-fold variation at District level).

**Conclusion:**

This is one of the largest studies of care home residents in England. We have identified factors associated with risk of residents having a high anticholinergic burden or sedative load which could be used to help target personalised interventions.

## Introduction

The harms associated with anticholinergic and sedative medication prescribing in older people (aged 65 and over) are well established including falls, cognitive decline, delirium, and hospitalisation.^1–4^ Risk of harm associated with anticholinergic and sedative medications is commonly measured using anticholinergic burden and sedative load scales. Although there are multiple scales with different weightings for medications, increased scores across different scales have been shown to be associated with increased risk of harm.^2^,^3^ Older people living in care homes may be at increased risk of these harms due to a higher prevalence of frailty and cognitive impairment.^5^,^6^

Despite limited evidence for the effectiveness of interventions aimed at reducing anticholinergic burden and associated harms in older people,^6^ the National Health Service (NHS) in England has produced recommendations and incentives aimed at reducing anticholinergic and sedative prescribing in this group. This includes performing structured medication reviews with validated anticholinergic burden scales and deprescribing of anticholinergic and sedative medications in patients with cognitive impairment.^7–10^ There is regional variation in how structured medication reviews are commissioned and performed within the wider Enhanced Health for Care Homes programme that NHS England introduced in 2019 to improve outcomes for care home residents and the focus has been on anticholinergic prescribing.^11^

There are limited studies that estimate anticholinergic burden and sedative load in care home residents in a UK setting or identify factors associated with higher rates of prescribing. A 2015 review assessing anticholinergic burden in the “oldest old” identified only two studies conducted in the UK (out of 13 included studies), both conducted in populations admitted to hospital.^5^ Subsequent studies have been conducted on regional populations of care home residents.^12^,^13^ No previous studies have used a national sample of older residents in England or measured variation in prescribing at a regional level that reflects how services aimed at reducing anticholinergic and sedative prescribing are commissioned.

We therefore aimed to: 1) Measure and identify factors associated with increased anticholinergic burden and sedative load in a national sample of older English care home residents; and 2) measure case-mix adjusted regional variation in anticholinergic burden and sedative load.

## Materials and Methods

### Study Design

This is a retrospective cohort study using routinely collected data derived from the Optimum Patient Care Research Database (OPCRD).^14^ We adhered to REporting of studies Conducted using Observational Routinely collected health Data Statement guidance.^15^ This forms part of a programme of research (UVAC study NIHR303605).^16^

### Setting

In England, health and social care services are commissioned by regional organisations called Integrated Care Boards (ICBs). At the time of the study there were 42 ICBs responsible for planning and performance of NHS services including some enhanced services for care homes (some are jointly commissioned with local authorities). The term “care home” is an umbrella term encompassing settings where people live and receive care and support. This includes care homes without nursing (residential care homes) that provide personal care delivered by care staff, with nursing care provided by community nursing teams, and care homes which provide personal and 24-hour nursing care (nursing homes). OPCRD provides de-identified routinely collected data for over 1100 UK General Practice (GP) providers (around 35% population coverage). GP providers are organisations which provide primary care to registered patients.

### Data Set

#### Primary Care Data

OPCRD identified residents within their dataset with either READ or SNOMED coding (Supplementary Material 1) indicating they were a care home resident at the beginning (index date) of four 6-month periods between 01/01/2023 and 31/12/2024. READ and SNOMED codes are a standardised coded thesaurus of patient findings and procedures.^17^ Prescription of anticholinergic and sedative medication included in the anticholinergic cognitive burden scale (most used measure in the NHS) and sedative load score in the following six months were estimated using recorded primary care prescriptions.^1^,^18^,^19^ This reflects the cumulative score for all anticholinergic and sedative medications prescribed in each 6-month period. Included medications are presented in Supplementary Material 2 with corresponding anticholinergic burden and sedative load scores. The number of medications included in these scores and corresponding scale values for each 6-month period were summed up for each resident and aggregated. This created four cohorts with anticholinergic burden and sedative load estimated in the 6-months following index date: Cohort 1 (01/01/2023 to 30/06/2023), Cohort 2 (01/07/2023 to 31/12/2023), Cohort 3 (01/01/2024 to 30/06/2024), and Cohort 4 (01/07/2024 to 31/12/2024).

Demographic information was extracted at each index date. Deprivation decile was estimated using the postcode of registered GP practice. Cambridge Comorbidity Index was calculated using all diagnoses recorded prior to index date, as was the electronic frailty index.^20^,^21^ The electronic frailty index, Sedative Score, and Anticholinergic Cognitive Burden were categorised using cut-offs validated in previous literature and used clinically.^1^,^22–24^ Based on previous literature, presence of urinary catheter was identified by coding 12-months, and feeding tube on coding 24-months, prior to index dates.^25^

#### Care Quality Commission Data

The 03/03/2025 CQC register of health and social care organisations in England was downloaded. Duplicate organisations were removed, as were organisations not registered as care homes with services for older people. Care home addresses were then used to cluster care homes by the postcode district (first part of UK postcode, 2302 postcode districts in England and Wales, average population 25,889 inhabitants per postcode district).^26^ Postcode districts were then clustered by ICBs using Office for National Statics data for ICB coverage (where districts encompassed multiple ICBs they were allocated to the ICB that covered the majority of postcodes within the district).^27^ Using the CQC register, the number and characteristics of care homes within a postcode district were estimated. An estimate of the total number of residents per district and ICB was made by aggregating the number of beds per care home in each district and ICB.

#### Data Linkage

Figure 1 shows how OPCRD and CQC data were clustered to create a multilevel structure and linked using postcode district. We excluded postcode districts and ICBs where less than 10% of care home residents had been sampled (total number of residents estimated by aggregating care home sizes). The Skills for Care adult workforce estimates by ICB were used to estimate percentage of care home jobs filled.^28^

**Figure 1:**
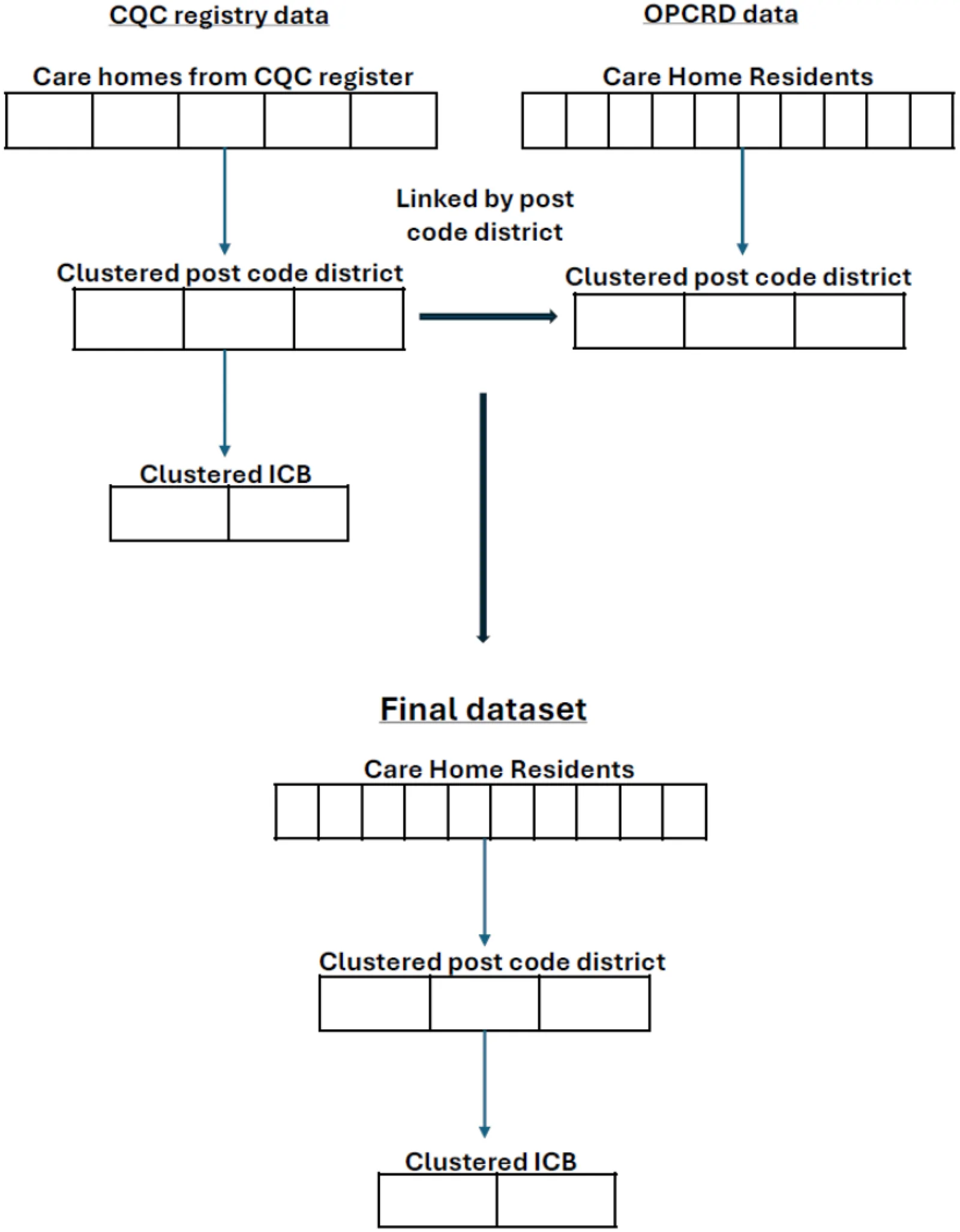
Data linked between CQC register and primary care data sets.

### Analysis

Descriptive characteristics of residents, care homes within districts, and care homes within ICBs were estimated. Residents with incomplete demographic information were excluded from analysis, as were residents in postcode districts not linkable to the CQC register. Anticholinergic cognitive burden and sedative load scores were dichotomised to produce binary outcomes (anticholinergic cognitive burden score >2 and sedative load >2) based on clinically used thresholds to indicate risk of adverse outcomes.^9^,^22^ A three-level (resident, district, and ICB) logistic regression model incorporating random intercepts predicting significant anticholinergic burden and sedative load per resident for the first 6-month period was estimated using the Sata melogic function.^29^ Random slopes were not specified in our models as our primary objective was to adjust for clustering across districts and ICBs. Predictors were included based on the findings of three previous systematic reviews, and advice from our advisory and patient and public involvement groups.^25^,^30^,^31^ Fractional polynomials were fitted to test for non-linear forms for all continuous variables using the Stata mfp function. Ranked caterpillar plots of postcode district and ICB effects with associated 95% confidence intervals were estimated. The analysis was repeated for the following 6-month periods with recalibration of predictor variables.

For each 6-month period, the expected number of patients with a significant anticholinergic burden and sedative load per district and ICB based on resident and care home characteristics was estimated using the logistic regression model. To identify outliers with higher or lower than expected rates of anticholinergic and sedative prescribing, funnel plots were produced of the ratio of observed to expected number of residents with a high anticholinergic burden and sedative load plotted against expected rates of prescribing. The 95% and 99.9% control limits were estimated using a random effects model to allow for overdispersion and we applied a trimming approach (excluding the top and bottom 10% of districts/ICBS that may unduly influence the limits).^32^,^33^ Analysis was completed using Stata version 15.1 and R version 4.5.2.

### Ethics

OPCRD has NHS HRA Research Ethics Committee approval to provide anonymised research datasets for research (NHS HRA REC ref: 20/EM/0148). The Anonymised Data Ethics and Protocol Transparency Committee approved use of the anonymised data for this project (ADEPT0325).

### Patient Public Involvement and Engagement (PPIE)

Patients and carer groups have supported study design, particularly advising on inclusion of predictor variables and outcomes. Our PPIE groups encompassed diverse perspectives, including professionals with experience working in care homes, care home residents, their family, and carers.

## Results

### Population Characteristics

Supplementary Material 3 shows the derivation of care homes clustered by postcode district and ICB from the CQC register. Supplementary Material 4 shows the derivation of the four 6-month care home resident cohorts clustered by districts and ICBs. Cohorts included between 49,174 and 51,155 residents clustered by between 448 and 474 postcode districts, and 32 to 35 ICBs. Figure 1 shows how these data sets were linked by postcode district. Table 1 shows the characteristics of residents and Table 2 shows postcode district and ICB level characteristics.

**Table 1:** Characteristics of Care Home Resident Cohorts

Population Characteristic	Level	01/01/2023- 30/06/2023 N=41,480	01/07/2023- 31/12/2023 N=43,508	01/01/2024- 30/06/2024 N=43,667	01/07/2024- 31/12/2024 N=44,705
Age	Median (IQR) Mean (SD)	86 (79–92) 85.1 (8.3)	86 (79–91) 84.7 (8.2)	86 (79–91) 85 (8.3)	86 (79–91) 84.7 (8.2)
Gender	Female	27,792 (67%)	28,982 (66.6%)	28,967 (66.3%)	29,629 (66.3%)
Ethnicity	White (British, Irish other)	36,377 (87.6%)	38,073 (87.5%)	37,829 (86.6%)	38,140 (85.3%)
	Asian/Asian British	305 (0.7%)	332 (0.8%)	345 (0.8%)	337 (0.8%)
	Black/Black British	148 (0.4%)	158 (0.4%)	176 (0.4%)	169 (0.4%)
	Hispanic	116 (0.3%)	126 (0.3%)	132 (0.3%)	126 (0.3%)
	Middle Eastern/Middle Eastern British	29 (0.1%)	31 (0.1%)	32 (0.1%)	32 (0.1%)
	Mixed black/Black British	81 (0.2%)	70 (0.2%)	72 (0.2%)	67 (0.2%)
	Mixed Other	57 (0.1%)	65 (0.2%)	69 (0.2%)	74 (0.2%)
	Other	142 (0.3%)	144 (0.3%)	146 (0.3%)	143 (0.3%)
	Unknown/Prefer not say	4,250 (10.2%)	4,509 (10.4%)	4,866 (11.1%)	5,617 (12.6%)
Deprivation Index Decile	1 (most deprived)	4,036 (9.7%)	4,512 (10.4%)	4,621 (10.6%)	4,628 (10.4%)
	2	6,138 (14.8%)	6,477 (14.9%)	6,718 (15.4%)	6,714 (15%)
	3	5,039 (12.1%)	5,170 (11.9%)	5,386 (12.3%)	5,460 (12.2)
	4	4,495 (10.8%)	4,537 (10.4%)	4,105 (9.4%)	4,283 (9.6)
	5	3,641 (8.8%)	4,017 (9.2%)	3,969 (9.1%)	3,984 (8.9%)
	6	4,753 (11.5%)	4,755 (10.9%)	4,839 (11.1%)	5,258 (11.8%)
	7	4,162 (10%)	4,262 (9.8%)	3,998 (9.2%)	3,967 (8.9%)
	8	2,470 (6%)	2,500 (5.8%)	2,667 (6.1%)	2,694 (6%)
	9	4,356 (10.5%)	4,624 (10.6%)	4,792 (11%)	4,926 (11%)
	10 (least deprived)	2,415 (5.8%)	2,654 (6.1%)	2,572 (5.9%)	2,791 (6.2%)
Electronic Frailty Index	Fit < 0.12	2,604 (6.3%)	2,638 (6.1%)	3,003 (6.9%)	3,355 (7.5%)
	Mildly Frail < 0.24	7,857 (18.9%)	7,887 (18.1%)	8,218 (18.8%)	8,394 (18.8%)
	Moderately Frail < 0.36	12,716 (30.6%)	13,257 (30.5%)	13,007 (29.8%)	13,198 (29.5%)
	Severely Frail	18,328 (44.2%)	19,726 (45.3%)	19,439 (44.5%)	19,758 (44.2%)
Cambridge Multi-morbidity Score	Median (IQR) Mean (SD)	3.4 (2.1–4.6) 3.4 (1.9)	3.4 (2.1–4.7) 3.4 (1.9)	3.4 (2.1–4.7) 3.4 (1.9)	3.4 (2–4.7) 3.4 (1.9)
Feeding tube	Present	210 (0.5%)	207 (0.5%)	206 (0.5%)	201 (0.5%)
Long term Catheter	Present	891 (2.2%)	918 (2.1%)	892 (2%)	955 (2.1%)
Resident-level Outcomes (6-monthly)					
Death	Yes	6,039 (14.6%)	5,527 (12.7%)	5,950 (13.6%)	3,676 (8.2%)
Anticholinergic cognitive burden score >2	Yes	9,619 (23.2%)	10,115 (23.3%)	10,173 (23.3%)	10,278 (23.3%)
Sedative load > 2	Yes	12,348 (29.8%)	12,789 (29.4%)	12,790 (29.3%)	12,906 (28.9%)

**Table 2:** Postcode District and Integrated Care Board (ICB) Characteristics

District					
District Characteristic	Level	01/01/2023- 30/06/2023 N=448	01/07/2023- 31/12/2023 N=461	01/01/2024- 30/06/2024 N=461	01/07/2024- 31/12/2024 N=474
Number Residents (estimated by number of care home beds)	Median (IQR)	75 (43–125)	78 (44–126)	79 (44–126)	78.5 (42–127)
	Mean (SD)	92.6 (71.6)	94.4 (73.8)	94.7 (74.8)	94.3 (74.9)
Number Care homes	Median (IQR)	6 (3–9)	6 (3–9)	6 (3–9)	6 (3–9)
	Mean (SD)	6.5 (4.4)	6.5 (4.4)	6.4 (4.4)	6.5 (4.4)
Mean size care home (number of beds)	Median (IQR)	39 (31.6–47.8)	39 (31.5–47.7)	39.6 (31.6–48.3)	39.3 (31.7–48)
	Mean (SD)	39.8 (13.1)	39.7 (13.3)	40.3 (13.9)	40.2 (13.4)
Proportion total care home bed capacity postcode included in cohort	Median (IQR)	37% (21.8–57.9%)	37% (22.2–58.5%)	37.3% (22.2–57.9%)	36.5% (20.5–56.7%)
	Mean (SD)	42.9% (30.7%)	43.1% (27.6%)	43.6% (29%)	42.1% (27.2%)
Percentage Care homes nursing	Median (IQR)	33% (16–55.6%)	33% (15–55.6%)	33% (15%-50)	33% (16.7%-55.6)
	Mean (SD)	37% (27%)	36% (27%)	36.4% (27.3%)	36.6% (26.9%)
Percentage Care homes with dementia care	Median (IQR)	75% (58–100%)	75% (58–100%)	75% (60–100%)	75% (60–100%)
	Mean (SD)	72% (24%)	72% (24%)	73.6% (23.5%)	72.7% (23.9%)
Mean care home rating 1: Inadequate 2: Requires Improvement 3: Good 4: Outstanding	Median (IQR)	2.8 (2.7–3)	2.8 (2.7–3)	2.8 (2.7–3)	2.8 (2.7–3)
	Mean (SD)	2.8 (0.28)	2.8 (0.28)	2.8 (0.28)	2.8 (0.27)
Number of ICBs covering district	1	346 (76.8%)	350 (75.9%)	349 (75.7%)	358 (75.5%)
	2	86 (19.2%)	91 (19.7%)	91 (19.7%)	95 (20%)
	3	17 (3.8%)	19 (4.1%)	20 (4.3%)	20 (4.2%)
	4	1 (0.2%)	1 (0.2%)	1 (0.2%)	1 (0.2%)
District-level Outcomes (6-monthly)					
Anticholinergic cognitive burden score >2	Median (IQR)	28 (17–45)	30 (18–46)	29 (17–46)	29 (16–46)
	Mean (SD)	34.8 (25.5)	35.1 (23.9)	35.7 (25.9)	35.4 (26.3)
Sedative load > 2	Median (IQR)	37 (22–59)	39 (23–56)	38 (22–58)	38 (22–58)
	Mean (SD)	44.4 (30.7)	44.1 (29.2)	45 (32)	44.4 (31.5)
Integrated Care Board (ICB)					
Integrated Care Board (ICB) Characteristic	Level	01/01/2023- 30/06/2023 N=32	01/07/2023- 31/12/2023 N=33	01/01/2023- 30/06/2023 N=34	01/01/2024- 30/06/2024 N=35
Number Residents (estimated by number of care home beds)	Median (IQR)	1135.5 (900–1590)	1128 (890–1575)	1098 (750–1572)	1087 (763–1502)
	Mean (SD)	1388 (894.5)	1403.3 (911.9)	1354.4 (943.8)	1348 (907.7)
Proportion total care home bed capacity ICB included in cohort	Median (IQR)	19.1% (12.6–22%)	20.6% (13.3–22.6%)	19.9% (13.5–22.6%)	20.7% (13.9–23.9%)
	Mean (SD)	18.2% (5.8%)	19.2% (6.1%)	19.5% (6.8%)	20.2% (7%)
Proportion jobs care home filled	Median (IQR)	95.8% (94.4–96.1%)	95.8% (94.4–96.1%)	95.8% (94.4–96.1%)	95.8% (94.3–96.1%)
	Mean (SD)	95.4% (1.6%)	95.5% (1.6%)	95.4% (1.4%)	95.4% (1.7%)
ICB-level Outcomes (6-monthly)					
Anticholinergic cognitive burden score >2	Median (IQR)	357 (237–525)	374 (228–514)	357 (249–524)	338 (222–534)
	Mean (SD)	423.9 (241.4)	436.5 (265)	439.9 (267.2)	421.8 (260.8)
Sedative load > 2	Median (IQR)	453 (309–684)	461 (308–674)	457 (305–683)	431 (275–681)
	Mean (SD)	540.5 (290.7)	546 (312.1)	553.5 (330.2)	533.6 (329.2)

The mean age of residents across cohorts was 85 years and around 66% of residents were female. There were high rates of frailty (>70% classified moderately or severely frail by electronic frailty index). On average postcode districts contained 6 care homes, of which 33% were nursing homes, and 75% had dementia care facilities. Around 3.5% of postcode districts contained more than 15 care homes and 6.5% contained one care home.

### Predictive Factors

The sizes of statistical association with residents having an anticholinergic cognitive burden score or sedative load > 2 for factors included in the 3-level logistic regression models are shown in Table 3 and Table 4, alongside the residual interclass correlation for each level (ICB and District), and C-statistic (measure of model discrimination and equivalent to area under the Receiver Operating Characteristic curve). The functional forms of included fractional polynomials are presented in Supplementary Materials 5 and 6. The ranked district and ICB level effects are presented in Supplementary Materials 7 and 8. Significant predictors of anticholinergic burden across all four time periods included age, male gender (reduced likelihood), Cambridge co-morbidity index, and frailty. An inverse relationship between age and likelihood of anticholinergic cognitive burden score > 2 was observed and likelihood of burden > 2 increased exponentially after a comorbidity index of 5 (Supplementary Material 5). Similarly, age, female gender, Cambridge co-morbidity index, and frailty also predicted a sedative load > 2. However, in addition to these predictors at a district level, the number of care homes and percentage of care homes with nursing care were associated with reduced and increased likelihood of a sedative load > 2, respectively.

**Table 3:** Strength of Association Between Factors Included in Logistic Regression Model and an Anticholinergic Cognitive Burden Score > 2

Care home resident level					
Population Characteristic	Unit change	01/01/2023- 30/06/2023 OR (95% C.I.)	01/07/2023- 31/12/2023 OR (95% C.I.)	01/01/2024- 30/06/2024 OR (95% C.I.)	01/07/2024- 31/12/2024 OR (95% C.I.)
(Age/10) ^−2^ - 0.0138106059*	1 year	3.77e+26 (1.17e+23 to 1.22e+30)	3.68e+28 (1.41e+25 to 9.58e+31)	1.98e+26 (8.02e+22 to 4.9e+29)	7.77e+25 (3.24e+22 to 1.86e+29)
Gender	Male	0.9 (0.88 to 0.97)	0.95 (0.9 to 0.998)	0.94 (0.89 to 0.99)	0.93 (0.89 to 0.98)
Ethnicity	White	Reference	Reference	Reference	Reference
	Asian	0.8 (0.6 to 1.01)	0.9 (0.7 to 1.2)	1.1 (0.8 to 1.4)	0.9 (0.7 to 1.2)
	Black	1.004 (0.7 to 1.5)	1.006 (0.7 to 1.5)	1.02 (0.7 to 1.5)	0.9 (0.6 to 1.4)
	Hispanic	0.7 (0.4 to 1.1)	0.8 (0.5 to 1.2)	0.7 (0.4 to 1.1)	0.9 (0.6 to 1.4)
	Middle Eastern	2.4 (1.1 to 5.2)	2.4 (1.1 to 5.1)	2.6 (1.2 to 5.7)	2 (0.9 to 4.4)
	Mixed black	1.1 (0.7 to 1.9)	1.3 (0.8 to 2.2)	1.1 (0.6 to 1.9)	0.9 (0.5 to 1.6)
	Mixed Other	0.7 (0.4 to 1.4)	0.7 (0.3 to 1.2)	0.8 (0.4 to 1.4)	0.9 (0.5 to 1.5)
	Other	1.2 (0.8 to 1.8)	1.3 (0.9 to 1.9)	1.1 (0.8 to 1.6)	1.1 (0.8 to 1.6)
	Unknown/Prefer not say	1.03 (0.9 to 1.1)	0.99 (0.9 to 1.1)	1.04 (0.96 to 1.13)	1.1 (1.03 to 1.2)
Deprivation Index Decile	1 (most deprived)	Reference	Reference	Reference	Reference
	2	1.05 (0.9 to 1.2)	1.09 (0.97 to 1.2)	0.99 (0.9 to 1.1)	1.03 (0.9 to 1.2)
	3	1.1 (0.99 to 1.3)	1.2 (1.1 to 1.4)	1.04 (0.9 to 1.2)	1.2 (1.1 to 1.4)
	4	1.01 (0.9 to 1.2)	1.1 (0.96 to 1.3)	1.02 (0.9 to 1.2)	1.2 (1.01 to 1.3)
	5	0.9995 (0.9 to 1.2)	1.1 (0.99 to 1.3)	1.2 (1.04 to 1.4)	1.3 (1.1 to 1.5)
	6	0.98885 (0.9 to 1.1)	1.05 (0.9 to 1.2)	0.98 (0.9 to 1.1)	1.02 (0.9 to 1.2)
	7	1.1 (0.9 to 1.3)	1.1 (0.9 to 1.3)	1.15 (0.99to1.33)	1.1 (0.95 to 1.31)
	8	1.1 (0.95 to 1.3)	1.2 (1.03 to 1.4)	1.3 (1.1 to 1.5)	1.3 (1.1 to 1.5)
	9	1.03 (0.9 to 1.2)	1.2 (1.02 to 1.4)	1.1 (0.98 to 1.3)	1.1 (0.9 to 1.2)
	10 (least deprived)	0.9 (0.8 to 1.1)	0.97 (0.8 to 1.1)	1.002 (0.8 to 1.2)	0.99 (0.8 to 1.2)
Electronic Frailty Index	Fit < 0.12	Reference	Reference	Reference	Reference
	Mildly Frail < 0.24	1.2 (1.1 to 1.4)	1.2 (1.05 to 1.3)	1.2 (1.03 to 1.29)	1.1 (1.01 to 1.3)
	Moderately Frail < 0.36	1.5 (1.3 to 1.7)	1.5 (1.3 to 1.6)	1.4 (1.2 to 1.5)	1.4 (1.2 to 1.5)
	Severely Frail	2 (1.7 to 2.2)	1.9 (1.7 to 2.2)	1.8 (1.6 to 2)	1.8 (1.6 to 2.)
Cambridge Multi-morbidity Score (CMI) In(CMI + 5.55111512313e-17)/10 + 1.078756129*	Unit increase	1.009 (1.004 to 1.01	1.008 (1.003 to 1.01)	1.01 (1.004 to 1.01)	1.01 (1.003 to 1.01)
Cambridge Multi-morbidity Score (CMI) (CMI + 5.55111512313e-17)/10)^3^–0.0393103126*	Unit increase	2.2 (1.8 to 2.7)	2.6 (2.2 to 3.1)	2.6 (2.2 to 3.2)	2.7 (2.2 to 3.2)
District Level					
(Number Care homes/10)^−2^-1.395903576	Care home	0.997 (0.995 to 0.999)	0.997 (0.994 to 0.999)	0.998 (0.996 to 1.0005)	0.997 (0.995 to 0.9996)
(Number Care homes/10)^3^–0.6063409095*	Care home	1.003 (0.995 to 1.01)	0.998 (0.99 to 1.007)	1.006 (0.997 to 1.0144)	1.001 (0.99 to 1.01)
Percentage Care homes nursing	Percentage	0.96 (0.8 to 1.2)	0.99 (0.8 to 1.2)	0.991 (0.996 to 1.0005)	0.9998 (0.8 to 1.2)
Percentage Care homes specialistic dementia	Percentage	0.9 (0.7 to 1.1)	0.9 (0.8 to 1.1)	0.99 (0.8 to 1.2)	0.9 (0.8 to 1.1)
Mean size care home	Care home bed	0.998 (0.993 to 1.002)	0.998 (0.99 to 1.002)	0.998 (0.995 to 1.002)	0.998 (0.994 to 1.003)
Mean care home rating	Mean CQC rating	1.08 (0.93 to 1.2)	1.03 (0.9 to 1.2)	0.99 (0.9 to 1.1)	0.97 (0.83 to 1.13)
Number ICBs district in	1	Reference	Reference	Reference	Reference
	2	1.1 (0.959 to 1.15)	0.998 (0.9 to 1.1)	1.01 (0.9 to 1.1)	1.08 (0.98 to 1.18)
	3	0.97 (0.8 to 1.2)	0.98 (0.8 to 1.2)	1.1 (0.9 to 1.3)	1.1 (0.9 to 1.3)
	4	0.96 (0.53 to 1.8)	1.1 (0.58 to 1.97)	0.9 (0.5 to 1.7)	1.3 (0.7 to 2.6)
ICC		0.033 (0.016 to 0.068)	0.03 (0.02 to 0.04)	0.03 (0.02 to 0.04)	0.03 (0.025 to 0.04)
ICB level					
(Proportion jobs care home filled×10) ^3^ −860.3173152*	Percentage	1.4 (1.02 to 1.9)	1.4 (0.97 to 1.9)	1.2 (0.7 to 2.2)	1.2 (0.9 to 1.7)
(Proportion jobs care home filled×10) ^3^× ln(Proportion jobs care home filled×10) −1937.807769*	Percentage	0.9 (0.8 to 0.99)	0.9 (0.8 to 1.01)	0.9 (0.7 to 1.1)	0.92 (0.81 to 1.05)
ICC		0.01 (0.005 to 0.02)	0.01 (0.007 to 0.2)	0.01 (0.006 to 0.2)	0.01 (0.006 to 0.2)
Overall Model Discrimination					
C-statistic		0.588 (0.581 to 0.594)	0.586 (0.579 to 0.592)	0.58 (0.57 to 0.59)	0.605 (0.5995 to 0.611)

**Notes**: *These formulae are fractional polynomial forms selected due to the non-linear relationship between the predictor and outcome. Forms are presented in Supplementary Material 5.

**Abbreviations**: ICB, Integrated Care Board; ICC, Intraclass Correlation Coefficient; OR, Odds Ratio.

**Table 4:** Strength of Association Between Factors Included in Logistic Regression Model and a Sedative Load Score > 2

Care home resident level					
Population Characteristic	Unit change	01/01/2023- 30/06/2023 OR (95% C.I.)	01/07/2023- 31/12/2023 OR (95% C.I.)	01/01/2024- 30/06/2024 OR (95% C.I.)	01/07/2024- 31/12/2024 OR (95% C.I.)
(Age/10)^3^–616.1421225*	1 year	0.972 (0.966 to 0.978)	0.971 (0.965 to 0.977)	0.976 (0.970 to 0.981)	0.974 (0.968 to 0.979)
(Age/10)^3^ *In(Age/10)-1319.258385*	1 year	1.011 (1.009 to 1.013)	1.011 (1.009 to 1.014)	1.009 (1.007 to 1.012)	1.01 (1.008 to 1.013)
Gender	Male	0.9 (0.8 to 0.9)	0.9 (0.8 to 0.9)	0.9 (0.8 to 0.9)	0.9 (0.8 to 0.9)
Ethnicity	White	Reference	Reference	Reference	Reference
	Asian	0.6 (0.5 to 0.8)	0.77 (0.6to0.99993)	0.8 (0.7 to 1.1)	0.6 (0.5 to 0.8)
	Black	0.7 (0.5 to 1.1)	0.9 (0.6 to 1.3)	0.8 (0.6 to 1.1)	0.7 (0.5 to 1.1)
	Hispanic	0.7 (0.5 to 1.1)	0.60 (0.37to 0.95)	0.5 (0.3 to 0.8)	0.6 (0.4 to 1.02)
	Middle Eastern	1.1 (0.5 to 2.5)	1.9 (0.9 to 4.1)	2.2 (1.1 to 4.7)	1.5 (0.7 to 3.1)
	Mixed black	0.8 (0.5 to 1.3)	0.8 (0.5 to 1.4)	1.04 (0.62to1.75	0.7 (0.4 to 1.2)
	Mixed Other	0.7 (0.4 to 1.3)	0.8 (0.4 to 1.4)	0.6 (0.4 to 1.1)	0.6 (0.4 to 1.1)
	Other	1.2 (0.8 to 1.7)	1.2 (0.8 to 1.7)	0.97 (0.67to 1.39)	1.1 (0.7 to 1.6)
	Unknown/Prefer not say	1.05 (0.97 to 1.13)	1.05 (0.98 to 1.14)	1.11 (1.04 to 1.20)	1.2 (1.1 to 1.3)
Deprivation Index Decile	1 (most deprived)	Reference	Reference	Reference	Reference
	2	1.06 (0.94to 1.19)	1.06 (0.94 to 1.2)	1.004 (0.89to1.1)	0.9 (0.8 to 1.04)
	3	1.06 (0.93to 1.2)	1.1 (0.9 to 1.2)	0.998 (0.9 to 1.13)	1.04 (0.9 to 1.2)
	4	0.99 (0.87to 1.13)	1.03 (0.9 to 1.17)	1.1 (0.9 to 1.2)	1.1 (0.9 to1.2)
	5	1.142 (0.996to1.31)	1.29 (1.12to 1.48)	1.3 (1.1 to 1.4)	1.2 (1.1 to 1.4)
	6	1.03 (0.89to 1.16)	1.01 (0.89to 1.16)	1.04 (0.9 to 1.2)	0.9 (0.8 to 1.1)
	7	1.03 (1.08to 1.18)	1.0002 (0.8642 to 1.1576)	0.98 (0.8 to 1.1)	0.97 (0.8 to 1.1)
	8	1.26 (1.08to 1.45)	1.3 (1.1 to 1.5)	1.3 (1.2 to 1.6)	1.1 (0.98 to 1.3)
	9	1.04 (0.9 to 1.19)	1.11 (0.96 to 1.28)	1.1 (0.9 to 1.2)	0.97 (0.8 to 1.1)
	10 (least deprived)	1.05 (0.89to 1.23)	1.09 (0.92 to 1.28)	0.97 (0.8 to 1.1)	0.89 (0.8 to 1.04)
Electronic Frailty Index	Fit < 0.12	Reference	Reference	Reference	Reference
	Mildly Frail < 0.24	1.2 (1.1 to 1.3)	1.2 (1.1 to 1.4)	1.1 (1.02 to 1.3)	1.1 (0.99 to 1.2)
	Moderately Frail < 0.36	1.3 (1.2 to 1.5)	1.3 (1.2 to 1.4)	1.2 (1.1 to 1.3)	1.1 (1.03 to 1.3)
	Severely Frail	1.5 (1.3 to 1.6)	1.4 (1.3 to 1.6)	1.4 (1.2 to 1.5)	1.3 (1.1 to 1.4)
Cambridge Multi-morbidity Score (CMI) In(CMI + 5.55111512313e-17)/10 +1.078756129*	Unit increase	1.41 (1.36 to 1.46)	1.46 (1.41 to 1.51)	1.4 (1.35 to 1.5)	1.46 (1.41 to 1.51)
Cambridge Multi-morbidity Score (CMI) In((CMI + 5.55111512313e-17)/10)^2^–1.163714786*	Unit increase	1.008 (1.007 to 1.009)	1.009 (1.008 to 1.010)	1.008 (1.007 to 1.009)	1.009 (1.008 to 1.010)
District Level					
(Number Care homes/10)^−1^-1.181483634*	Care home	0.96 (0.94 to 0.98)	0.96 (0.94 to 0.98)	0.97 (0.95 to 0.99)	0.97 (0.95 to 0.99)
Percentage Care homes nursing	Percentage	1.23 (1.04 to 1.46)	1.3 (1.1 to 1.6)	1.2 (1.0003 to 1.4)	1.2 (0.98 to 1.4)
Percentage Care homes specialistic dementia	Percentage	0.9 (0.8 to 1.1)	0.9 (0.8 to 1.1)	1.1 (0.9 to 1.3)	0.98 (0.8 to 1.2)
Mean size care home	Care home bed	1.002 (0.998 to 1.006)	1.001 (0.997 to 1.005)	1.001 (0.997 to 1.005)	1.001 (0.997 to 1.004)
Mean care home rating	Mean CQC rating	0.97 (0.85 to 1.12)	0.89 (0.77 to 1.03)	0.97 (0.8 to 1.1)	0.9 (0.8 to 1.1)
Number ICBs district in	1	Reference	Reference	Reference	Reference
	2	0.99 (0.90to 1.08)	0.97 (0.88to 1.06)	0.996 (0.9to 1.1)	1.05 (0.95 to 1.1)
	3	1.1 (0.9 to 1.3)	0.99 (0.83to 1.18)	1.1 (0.9 to 1.3)	0.96 (0.8 to 1.1)
	4	1.1 (0.6 to 2)	0.98 (0.51to 1.88)	1.1 (0.6 to 2)	1.2 (0.6 to 2.3)
ICC		0.02 (0.015 to 0.027)	0.03 (0.02 to 0.04)	0.026 (0.02 to 0.034)	0.029 (0.022 to 0.037)
ICB level					
Proportion jobs care home filled	Percentage	0.4 (0.02 to 8.9)	0.45 (0.01 to 20.06)	4.1 (0.05 to 365.8)	0.2 (0.004 to 5.9)
ICC		0.003 (0.001 to 0.009)	0.006 (0.003 to 0.014)	0.007 (0.003 to 0.01)	0.006 (0.003 to 0.1)
Overall Model Discrimination					
C-statistic		0.602 (0.596 to 0.608)	0.606 (0.601 to 0.612)	0.605 (0.6 to 0.611)	0.6 (0.59 to 0.61)

**Notes**: *These formulae are fractional polynomial forms selected due to the non-linear relationship between the predictor and outcome. Forms are presented in Supplementary Material 6.

**Abbreviations**: ICB, Integrated Care Board; ICC, Intraclass Correlation Coefficient; OR, Odds Ratio.

### Observed versus Expected Rates of Significant Anticholinergic Burden and Sedative Load

Figure 2 shows the funnel plots estimated of the ratio of observed to expected number of residents with an anticholinergic burden > 2 at ICB level and Figure 3 the ratio of observed to expected number of residents with a sedative load > 2 at ICB level. Equivalent funnel plots estimated for districts are shown in Supplementary Material 9 and 10.

**Figure 2:**
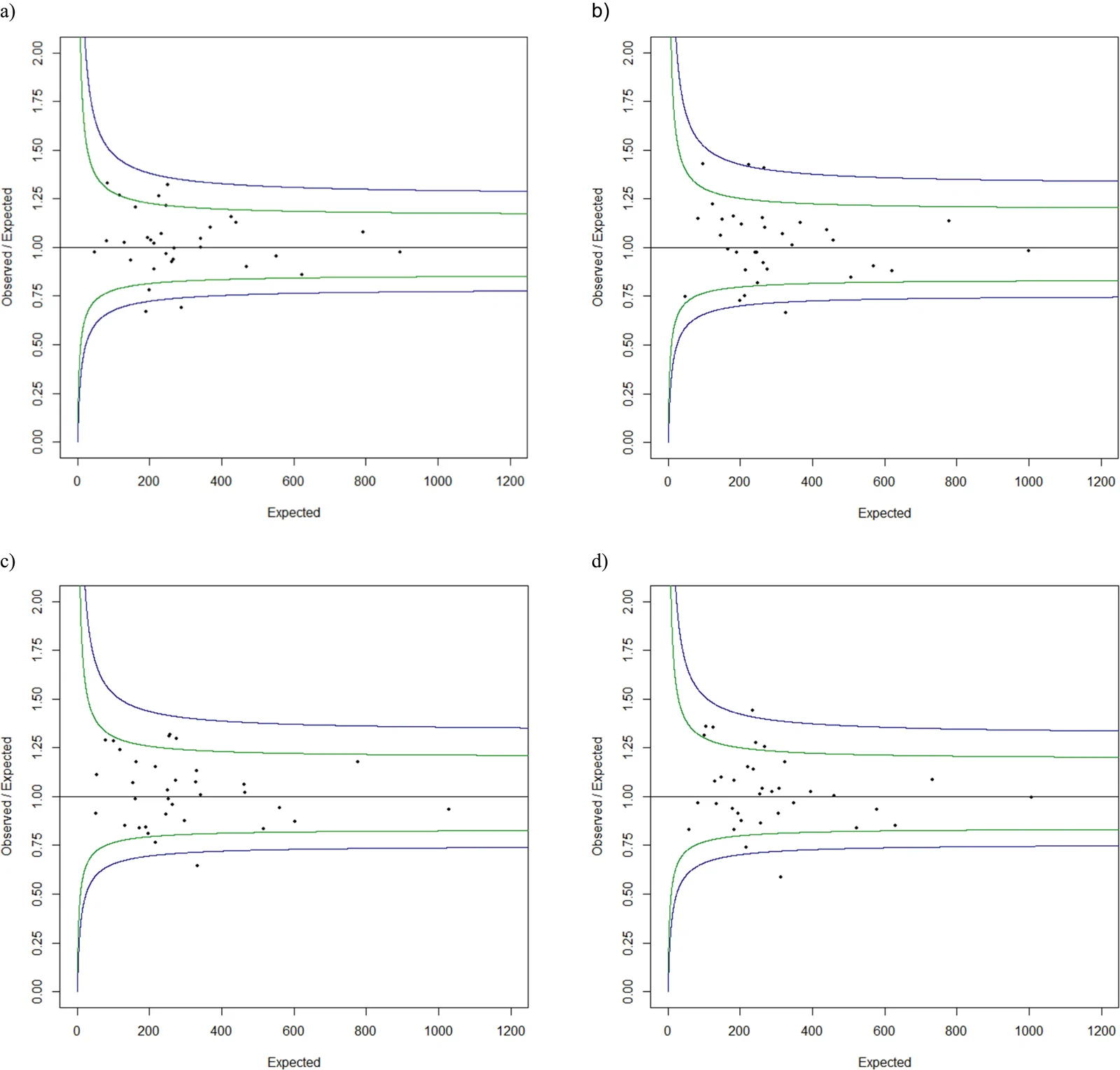
ICB level funnel plots of observed versus expected with an anticholinergic burden anticholinergic burden > 2. Green lines 95% control limits, blue lines 99.9% control limits. (**a**) Cohort 1 (01/01/2023 to 30/06/2023); (**b**) Cohort 2 (01/07/2023 to 31/12/2023); (**c**) Cohort 3 (01/01/2024 to 30/06/2024); (**d**) Cohort 4 (01/07/2024 to 31/12/2024).

**Figure 3:**
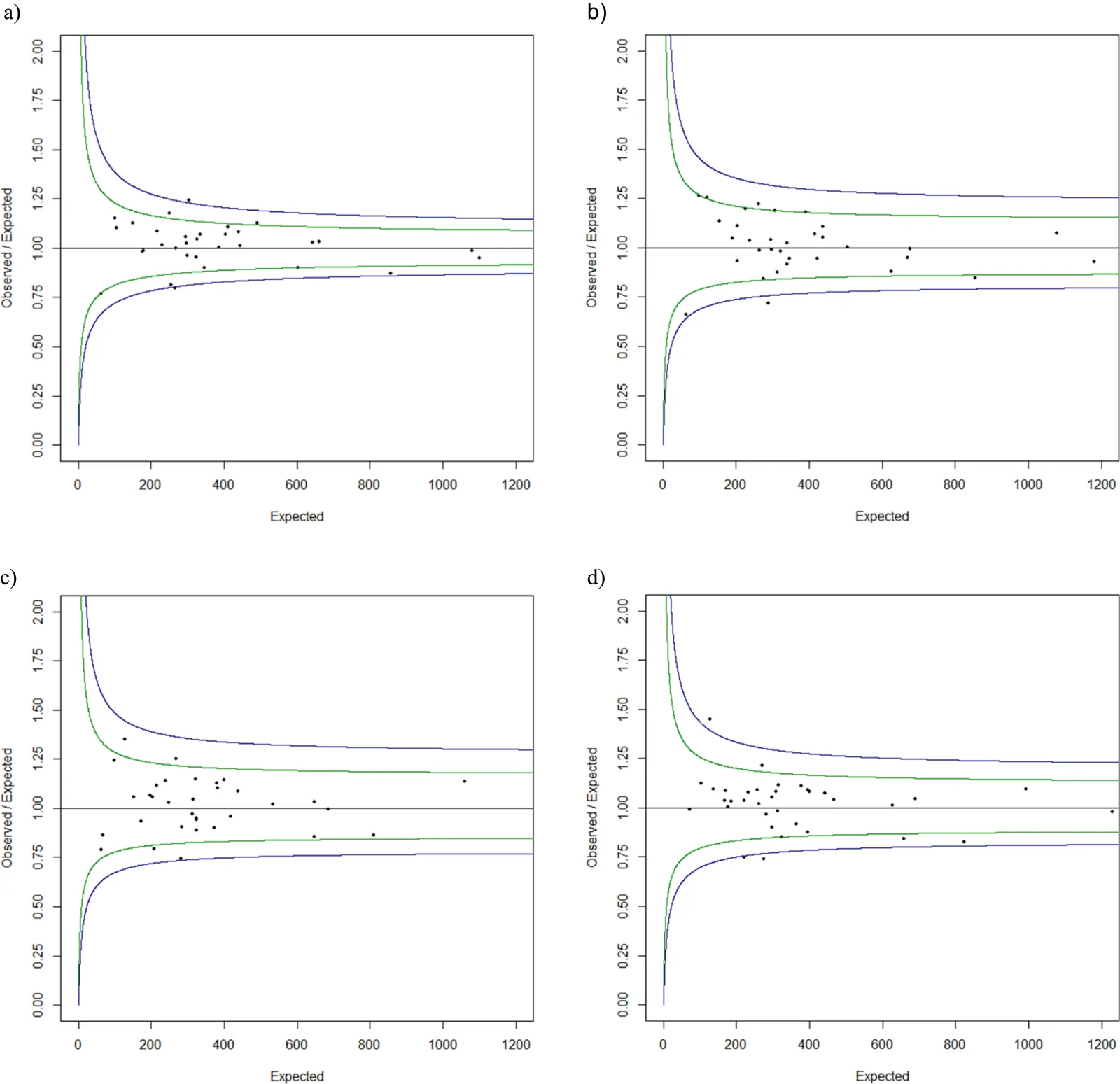
ICB level funnel plots of observed versus expected with a Sedative Load > 2. Green lines 95% control limits, blue lines 99.9% control limits. (**a**) Cohort 1 (01/01/2023 to 30/06/2023); (**b**) Cohort 2 (01/07/2023 to 31/12/2023); (**c**) Cohort 3 (01/01/2024 to 30/06/2024); (**d**) Cohort 4 (01/07/2024 to 31/12/2024).

We found some variation at ICB level in observed versus expected number of residents with an anticholinergic burden > 2. The same ICB was an outlier at a 95% control limit in all four 6-month periods and had between 1.3 and 1.4 times as many residents with an anticholinergic burden > 2 as would be expected. Conversely, another was an ICB outlier at 95% control limits in all four 6-month periods had between 0.6 and 0.7 times as many residents with an anticholinergic burden > 2 as would be expected. We found more variation at postcode district level. However, due to the larger number of included districts with small numbers of residents sampled in some districts, these results need to be interpreted more cautiously. An outlying district at a 99% control limit in all four 6-month periods with over 70 residents sampled in each period (around 20% of residents in district based on number of care home beds) had between 1.9 and 2.1 times as many residents with an anticholinergic burden > 2 as would be expected. Conversely, a district with around 50 residents sampled in each 6-month period (15% of total residents) was an outlier at a 95% control limit in all four 6-month periods with between 0.6 and 0.7 times as many residents with an anticholinergic burden > 2 as would be expected.

We found less variation at ICB and district level in observed versus expected number of residents with a sedative load > 2. The same ICB was an outlier at a 95% control limit in all four 6-month periods and had between 1.1 and 1.2 times as many residents with a sedative load > 2 as would be expected. Conversely, another was an ICB outlier at 95% control limits in all four 6-month periods and had between 0.7 and 0.8 times as many residents with a sedative load > 2 as would be expected. An outlying district at a 95% control limit in all four 6-month periods with over 30 residents sampled in each period (around 11% of residents in district based on number of care home beds) had between 1.4 and 1.9 times as many residents with a sedative load > 2 as would be expected. Conversely, a district with around 100 residents sampled in each 6-month period (15% of total residents) was an outlier at a 95% control limit in all four 6-month periods with between 0.7 and 0.9 times as many residents with a sedative load > 2 as would be expected.

## Discussion

### Summary

We have created four 6-month cohorts of approximately 20% of care home residents in England.^26^ Our logistic regression modelling (although only demonstrating poor levels of discrimination (C-statistic around 0.6)) found female gender, reduced age, increasing frailty, and increasing comorbidity to be associated with an anticholinergic cognitive burden and sedative load > 2. We compared observed number of residents with anticholinergic burden and sedative load score> 2 to those predicted based on case-mix at district and ICB levels. We demonstrated some case-mix adjusted variation at both ICB and postcode district level with up to 2.3-fold variation at ICB and 3.5-fold variation at district level in anticholinergic burden. We found up to 1.7-fold variation at ICB and 2.7-fold variation at District level in sedative load.

### Comparison to Previous Literature

Our sample appears representative of the care home population when compared with 2021 census data for England. In the census, the median age of residents was 86 years, around 66% of residents were female, and 97.5% of the population was white.^34^ Only a third of care homes were nursing homes but 49% of residents lived in nursing homes. Across our cohorts the median age was 86, around 67% were female, 87.6% were white (over 10% were unknown), and 33% of care homes were nursing homes. We were unable to determine if residents resided in residential or nursing homes in our data.

Our estimated proportion of care home residents with a high anticholinergic cognitive burden (anticholinergic cognitive burden score > 2: 23.3%) is higher than previous studies. Grill et al found around 12% of residents had an equivalent anticholinergic burden in 147 care homes in two health care boards in Scotland.^12^ However, this study assessed cumulative prescribing of medications within a 56 day time period, whilst we assessed cumulative prescribing within a 6-month period. One large review states that around 20% of older adults have problematic anticholinergic prescribing.^35^ A study using community prescribing data found that over 65% of adults aged over 65 in Ireland were prescribed at least one anticholinergic or sedative drug.^36^ We estimated around 29% of our care home population to have a sedative load > 2. This is similar estimate to that reported in a population level cross sectional study in Scotland which found 28.4% of care home residents were prescribed benzodiazepine or z-hypnotic medications.^37^ Previous studies, like ours, identified both younger age and comorbidity to be associated with higher risk of problematic prescribing.^12^ An association between comorbidities and age has also been found with poly-pharmacy and antimicrobial prescribing in cohorts of Scottish care home residents.^38^,^39^

We note that there is limited research identifying predictors of anticholinergic burden or sedative load in care home populations with most previous reviews focusing on different ways of classifying these medications, their association with adverse outcomes, or interventions to promote de-prescribing.^2^,^18^,^35^ We believe that is the largest cohort study in England to identify factors associated with anticholinergic and sedative prescribing and the first study to estimate risk-adjusted variation in prescribing at regional commissioning (ICB) or postcode district (service provide) level. However, Grill et al have estimated variation in prescribing at a care home level in a comparable Scottish population and based on intra-cluster correlation coefficient (ICC) found significant between care home variation in prescribing (hypnotics/anxiolytics ICC 7.3% and anticholinergics ICC 2.8%). Based on ICC, the amount of variation we identified at ICB and district level was smaller with 1% variation found at ICB and 3% variation at postcode district level in anticholinergic prescribing and <1% at ICB level and around 3% variation at postcode district level in sedative prescribing. Higher levels of variation at care home level have also been found for antimicrobial prescribing and polypharmacy in similar Scottish care home resident cohorts which may indicate that care home level factors are more important determinants of prescribing practice.^38^,^39^

### Strengths and Limitations

This is one of the largest cohort studies of care home residents in England. The use of primary care data linked to the CQC register allowed us to assess the predictive value of individual and organisational factors in combination.

We are making inferences at a population level using a 20% sample of care home residents in England and this may cause selection bias. The validity of these inferences depends on how representative our sample is of the national care home population and of residents in included postcode districts and ICBs. Our sample does appear representative of the national population when compared to census data, but there may be differences between residents registered with GP practices which submit to OPCRD and other residents. Comparison of methods for identifying care home residency in routine data found that identifying residents using READ and SNOMED coding may underestimate the number of residents compared with identification using linkage of address.^40^ Due to anonymisation, it was not possible for us to use address. However, as part of the Enhanced Health in Care Home Framework all care homes in England should now have a named primary care practice, increasing reliability of identification of residency using READ and SNOMED coding in primary care data.

Due to electronic prescribing within primary care within an NHS setting, prescribing of anticholinergic and sedative medications is reliably identified within our data. However, other factors included in our modelling may be less well recorded. In particular, ethnicity may be poorly recorded within routine data and deprivation index was estimated based on the primary care practice residents were registered with. Primary care practices may serve populations across a wide range of deprivation levels, and the practice-level deprivation may not reflect the deprivation experienced by the care home or residents. Individual comorbidities can also be recorded with some variability within the routine data collected within primary care. To mitigate this potential source of bias we used the Cambridge Comorbidity Scale and electronic frailty index which combines multiple individual diagnostic codes to predict patient outcomes; their use in research has been validated in UK primary care datasets.^21^ However, this prevented us from investigating whether individual co-morbidities are associated with risk of increased anticholinergic burden or sedative load. We were also unable to differentiate between residents living in care homes with or without nursing care at an individual level in the available data (although we conducted some adjustment for this using characteristics of care homes at district level). We were also unable to test the association of measures of health and social care resource allocation with prescribing practices.

### Implications

The associations between frailty and co-morbidity and increased risk of anticholinergic burden and sedative load are important because they are identifiable using routinely collected data and can inform personalised care plans formed as part of targeted interventions, such as those suggested in the Enhanced Health in Care Homes programme.^41^ However, model discrimination for our estimated models ranged from 0.58 to 0.61 and with correspondingly small associations between significant predictors and our outcomes. Therefore, the findings should not be used to inform clinical practice in isolation.

NHS service improvement work has had a greater focus on reducing anticholinergic prescribing than prescribing of sedatives. Although our observational study does not test the effect of such initiatives, it is possible that the lower rate of patients with high anticholinergic burden (23%) compared to those with a high sedative load (around 30%) reflects a greater NHS focus on reducing anticholinergic prescribing. The greater variation at both ICB and district level in risk adjusted rates of patients with an anticholinergic load > 2 compared to those with a sedative load > 2 may suggest that such service improvement work to reduce anticholinergic prescribing has not been uniformly implemented effectively. The greater level of variation demonstrated by both estimates of the ICC and our funnel plots at district compared to ICB level for both anticholinergic burden and sedative load indicates that any future interventions aimed at reduced anticholinergic and sedative prescribing need to be aimed at the level of individual care homes and health care providers at a postcode district level. This supports findings from studies assessing care home level variation in antimicrobial prescribing and polypharmacy.^38^,^39^

However, as argued by Jenks and Burton,^42^ although analysis using routine data to highlight variation in problematic prescribing in care homes is useful to highlight where prescribing practices could be improved, limitations in routine data prevent nuanced investigation into how this should be achieved. Two large cluster randomised control trials of care home level interventions to optimise prescribing in England found they were effective in reducing polypharmacy, but this did not translate into improved resident outcomes.^43^,^44^ As found in qualitative research conducted alongside these trials, interventions tailored to individual care home may be needed to address variation in anticholinergic, sedative, and other problematic prescribing.^45^

## Conclusion

We have conducted one of the largest cohort studies of care home residents in England. We have identified important factors associated with risk of residents having a high anticholinergic burden or sedative load which could be used to help target personalised interventions. We identified some variation in anticholinergic and sedative prescribing at commissioner level and more variation at provider level. Provider level variation may reflect unmeasured confounders (at individual or care home level) or variation in prescribing at a provider level. If identified provider level variation reflects differences in prescribing, future interventions aimed at reducing anticholinergic and sedative prescribing should be aimed at a provider level to have the greatest effect.

## Research Data Transparency and Availability

Data may be obtained from a third party and are not publicly available. The data used for this study are subject to data sharing agreement with Optimum Patient Care which prohibits further sharing of individual level data. The datasets used are obtainable from these organisations subject to necessary authorisations and approvals.
